# On Convulsion

**Published:** 1859-01-01

**Authors:** 


### Art. VI.—ON CONVULSION.

In ancient times, the prevalent idea was that a convulsed person was possessed and supernaturally excited by some good or bad spirit. It seemed as if the convulsion was not to be accounted for by any kind of natural power. And this idea, most assuredly, is one which derives not a little support from the text of the New Testament, where instances are mentioned in which convulsions of one kind or another were cured by the direct casting out of an evil spirit. In modern times a different explanation is offered, and "nervous influence," among other influences, is called upon to do the work of the wayward or malignant spirit of times past. The idea appears to be this:—Muscle is a living thing, and contraction is the outburst of this life. In other words, it is supposed that muscle is endowed with a vital property of contractility, and that muscular contraction is brought about when this property is put in action by certain agents, such as nervous influence and electricity. It is supposed, indeed, that this property of contractility is roused or excited or *stimulated* into a state of action when the muscle contracts under ordinary circumstances, and that this property is roused or excited or stimulated beyond measure in convulsion. In this, at least, the modern hypothesis agrees with the ancient opinion, that in each instance the convulsion is looked upon as the manifestation of additional life in the muscles,—a life foreign to the individual in the one case, an excess of life belonging to the individual in the other case.

The present, however, is an age of change, and among other things doomed to change, may be these opinions. At any rate, it is now maintained, and with no small show of reason, that muscular contraction is produced, *not* by the stimulation of any vital property of contractility belonging to muscle, but by the simple cessation of the action of certain agents—electricity and others—which had previously kept the muscle in a state of relaxation or expansion. It is maintained that convulsion in every form, and under every circumstance, is explainable in the same manner, and in this manner only. In a word, a physical and intelligible hypothesis is offered in place of a vital and unintelligible hypothesis—for to call a phenomenon vital, is to confess that it is unintelligible.

The hypothesis to which we here refer, and which has been advocated for several years by Dr. Radcliffe, has been brought out recently in a much more circumstantial and satisfactory form*—a form so satisfactory that we think it necessary to do what we can to make it known, for it is assuredly of high moment to all who are concerned in the study of diseases of the brain and nervous system to have right notions on the subject of convulsion.

Dr. Radcliffe begins his inquiry into the physiology and pathology of muscular action by showing that muscular contraction is *not* produced by the stimulation of any property of contractility belonging to muscle; and first of all he attempts to show that the action of electricity in muscular contraction is not that of a stimulus.

The principal facts under this head are these:—Muscle, in common with nerve and other, if not all, animal tissues, is the seat of definite electrical actions. These actions are only manifested in irritable and living muscle; they are proportionate to the degree of irritability and vitality; they die out as the signs of irritability and vitality disappear; and when these signs have disappeared, and the muscles have passed into the contraction of death, *rigor mortis*, they are altogether at an end. In ordinary contraction, also, there is an unmistakeable *weakening* of that electrical action which naturally belongs to muscle in the state of relaxation or elongation. This fact is very curious, and it is well to hear what Dr. Radcliffe has to say about it:—

"It would also seem as if the muscular current were *weakened* during ordinary contraction; indeed, there can be no reasonable doubt that this is the case. In demonstrating this very important fact, Dr. du Bois-Reymond makes use of the gastrocnemius of a frog with a long portion of its nerve attached. This muscle is placed upon the cushions of the galvanometer, and the nerve is arranged in such a manner that a series of shocks from an induction coil can be passed through a portion of it. As soon as the muscle is laid upon the cushions, the needle of the galvanometer is deflected to some distance from zero, and it continues to be deflected, though not to same extent, so long as the muscle remains in a state of rest; as soon as the muscle is tetanized by passing the interrupted current through a portion of the nerve, the needle immediately travels back again and oscillates on the other side of zero. These are the simple facts. As soon as the muscle is tetanized, that is to say, *the needle is acted upon by a reverse current.*

"Now it cannot be supposed that this current is due to the irruption of a current from the induction apparatus by which the muscle is tetanized into the circuit of the galvanometer, for both galvanometer and coil are carefully insulated. Moreover, there is the same reverse current

* "Epilepsy and other Convulsive Affections." Second Edition. Churchill, London. 1858.

(only in a less marked degree) when the muscle is tetanized by other means, as by subjecting the nerve to mechanical or chemical irritation, or by acting upon it by heat. Nay, the same current is seen to be produced by strychnine in a gastrocnemius which is still in living connexion with the nervous centres by means of the ischiatic nerve.

"Nor is the reverse current, which is manifested during tetanus, to be regarded as a proof that there is a muscular current during contraction, whose direction is contrary to that of the current which plays during the state of rest. On the contrary, it is possible that the muscular current, before the state of tetanus was induced, may have given rise to a reverse current within the circuit of the galvanometer by evolving the secondary polarity of the platinum plates, and that this reverse current may be manifested during tetanus, in consequence of the proper muscular current having become *weakened*. And that this is the true explanation may be seen by the following modification of the same experiment.

"The only difference between the experiment as modified and the original experiment is this—that one of the electrodes, instead of being connected with the galvanometer by a continuous piece of wire, is connected by a broken wire, of which the two ends are made to dip into a small cup of mercury. The difference, that is to say, is in a simple contrivance for rapidly breaking or closing the circuit; for it must be plain that the circuit is broken when an end of the wire is raised out of the mercury, and closed when the same end is replaced in the mercury. In performing the modified experiment, the muscle is first laid upon the cushions, and a note made of the degree to which the needle of the galvanometer is deflected. Then the muscle is removed from the circuit of the galvanometer, and the instrument depolarized by raising one end of the wire out of the mercury. In the next place, the muscle is tetanized, and while it is in this state it is included in the circuit of the galvanometer by replacing the end of the wire in the mercury. In this way, then, the current of the tetanized muscle is for an instant separated from the reverse current of secondary polarity, which is caused by the continued action of any current upon the instrument, and the result is that the needle travels in the *same* direction as that in which it travels under the current of the untetanized muscle, *but not to the same distance from zero.* In other words, the muscular current of the tetanized muscle is found to be *weakened*, but not changed—as it would appear to be if no care be taken to eliminate the influence of secondary polarity from the experiment. In several instances in which I repeated this experiment upon the ordinary frog, the primary deflection of the needle, under the current of the elongated muscle, was from 40° to 60°, and the permanent deflection from 5° to 7°; whereas the primary deflection of the needle, under the current of the tetanized muscle, was from 8° to 15°, and the permanent deflection from 1° to 2°."—(pp. 26-30).

Here, then, are two facts to begin with—two facts which are of primary and fundamental importance. The one is, that the contraction of death, *rigor mortis,* does not come on until the electrical action of muscle—or the muscular current, as it is called—is at an end; the other is, that the muscular current is weakened in ordinary muscular contraction. These are the facts; the inference arising out of them, according to Dr. Radcliffe, is, that muscular contraction is *antagonized* by the muscular current.

Nor is a different inference deducible from the action of artificial electricity upon muscle. For what are the main facts? The first fact is, that, except at the moment when the circuit is made and broken, the muscle is kept in a state of relaxation or elongation during the whole time that a constant electrical current is made to pass through it. The second fact is, that a contracted or tetanized muscle is relaxed or elongated by the passage of a constant current through it. But for the contractions which attend upon the moment when the circuit is made and broken, the facts, indeed, would seem to show, if they show anything, that muscular contraction is antagonized rather than caused by the action of the foreign electrical current upon muscle. Nay, the contractions which attend upon the moment when the foreign current begins and ceases to pass, may be no objection to this view; for at the time when these contractions happen, there may be a momentary annihilation of electrical action arising out of certain opposing reactions between the foreign and muscular currents, which reactions are very clearly pointed out by Dr. Radcliffe, but which would take us too long to explain in this place.

In the next place, the object is to show that muscular contraction is not produced by "nervous influence." Here, first of all, Dr. Radcliffe speaks of the nerve current—which current is an unquestionable fact in physiology—and shows that it agrees with the muscular current, in that it affords evidence of enfeeblement during muscular contraction. Take a beautiful experiment by Dr. du Bois-Reymond as an instance in point:—

"A frog is fastened upon a suitable frame, and then, after tying its common iliac artery, the ischiatic nerve is cut low down in the ham and dissected up to the vertebral column. After this the lower end of the nerve is bridged over the cushions of the galvanometer, so as to touch one cushion with its end and the other cushion with its side, and a note is taken of the degree to which the needle of the galvanometer is deflected by the 'nerve current.' The animal is then poisoned with two or three drops of a solution of nitrate of strychnia, and the effect of the tetanus upon the 'nerve current' is attended to by watching the movements of the needle of the galvanometer. The experiment is simple and the result unmistakeable; for when the tetanus occurs, *the needle recedes three or four degrees nearer to zero*, and this not only during the principal attacks, but also during those single shocks which are produced on touching the animal. It is seen further that the needle continues at this point so long as the tetanus continues, and that it again diverges from zero when the spasm passes off. It is seen, indeed, that the 'nerve current' is enfeebled during muscular contraction. In some frogs upon which I repeated this beautiful experiment, the primary deflection of the needle under the nerve current *before* the supervention of the tetanic symptoms was from 15° to 20°, and the permanent deflection from 2° to 4°; and, *after* the supervention of these symptoms, the primary deflection was from 3° to 4°, and the permanent deflection scarcely perceptible." (pp. 44, 45.)

With respect to the action of a foreign current upon the nerve, it is also found that the results, so far as muscular action is concerned, are precisely analogous to those which attend the action of this current upon muscle, in that, except at the moment when the current begins and ceases to pass, a relaxed muscle remains relaxed, and a contracted or tetanized muscle relaxes and then remains relaxed, so long as a constant current is made to pass through a living nerve. The contractions attending upon the time when the current begins and ceases to pass, are also referred to a momentary annihilation of electrical action, arising out of certain opposing reactions between the nerve current and foreign current, which reactions are clearly pointed out; and not only so, but an apparent explanation is found, in certain intelligible differences of these reactions, of the reason why the degree of contraction at the commencement and cessation of the current is different when the current is said to be inverse, and when it is said to be direct,—and also of those curious revivals of contraction on changing the direction of the current which are known as *voltaic alternatives*. For these matters, however, we must refer to the work itself.

In short, it would seem as if muscular elongation is coincident with the *action* of natural or foreign electricity upon the nerve, and that muscular contraction is connected in some way with the diminution or annihilation of this action, and not with the stimulating action of electricity upon a vital property of irritability inherent in nerve.

After these considerations, Dr. Radcliffe adduces facts which seem to demolish the idea that muscular contraction is produced by any kind of stimulation derived from the nervous centres. Thus:—

"In these experiments, weights of different sizes are suspended from a small hook that has been previously attached to one of the hind legs, a little above the heel. The frog is then held up by its fore limbs, and a weight that is just sufficient to put the hind leg gently upon the stretch is placed upon the hook. In the next place the toe of the weighted leg is pinched, and the weight is changed for one heavier, until the animal is no longer able to withdraw its leg from the torture to which it is subjected. This being done, the next thing is to divide the spinal cord immediately behind the second pair of nerves; and to go on testing the muscular power of the paralysed legs. The results are very strange. Immediately after the operation, the muscular power that can be put forth by the weighted leg when the toe is pinched is sometimes nil, but generally it is no more than a third or fourth of what it was before the operation. Fifteen minutes later this power is evidently rallying. In twenty or five-and-twenty minutes it has recovered all it had lost. An hour after the operation it is greater than it was before the operation—perhaps doubled. An hour or two later still, it is certainly doubled, and possibly trebled; and from this time up to the twenty-fourth hour, when the increase generally attains its maximum, it goes on slowly augmenting. The particulars of two experiments with very fine frogs (A and B) were as follows, the weights raised being expressed in grammes:

| | Before the Operation. | Immediately afterwards. | 5 minutes afterwards. | 15 minutes afterwards. | 25 minutes afterwards. | 1 hour afterwards. | 2 hours afterwards. | 4 hours afterwards. | 24 hours afterwards. | 48 hours afterwards. |
|---|---|---|---|---|---|---|---|---|---|---|
| A | 60 Gram. | 20 | 45 | 60 | 80 | 130 | 140 | 140 | 150 | 150 |
| B | 60 Gram. | 10 | 30 | 40 | 60 | 100 | 120 | 130 | 140 | 140 |

"When the increase of the muscular power has attained its maximum, it may remain nearly stationary for six, ten, fifteen, or twenty days, and after this time it fails by slow degrees. In a month, if the animal lives, this power will have fallen to its original value before the operation; and at the expiration of six, seven, or eight months, it may have fallen still lower, until it is not more than a third or a half of this value. It is possible, however, that the increased muscular power would not have failed in this way if care had been taken to exercise the paralysed limbs by galvanism.

"Nor is it easy to agree with Dr. Marshall Hall in thinking that this increase of muscular power is due to an increased stimulation of the muscles on the part of the spinal cord (which inordinate stimulation had come into play because the controlling influence of the brain had been withdrawn), for there are other experiments which show plainly enough that the muscular power is augmented, not only in a similar but even in a higher degree, after the muscle has been cut off altogether from the spinal cord.

"In the paper on muscular irritability, to which reference has already been made, and which is deserving of study, as well for the facts as for the opinions contained in it, Professor Engel, of Zurich, has shown that muscles are more prone to enter into the state of contraction after the complete removal of the cerebro-spinal system. In this experiment he clips out the whole of this system, bones and all, and, after five or ten minutes, he finds that the muscles have become so irritable as to be thrown into a state of contraction by a blow on the table. He finds, indeed, that the muscles are as irritable as they are in narcotized frogs.

"Some very conclusive evidence to the same effect is also furnished in the following experiment by Dr. Brown-Séquard. In this experiment the spinal cord of a frog is divided immediately behind the roots of the brachial nerves, and then the nerves proceeding to *one* of the hind legs, cut through at the points where they leave the cord. *Two hours later both the hind limbs are separated from the body*, and the contractility of their muscles is tested by the prick of a needle and by galvanism. This is the experiment. The result is, *that the 'irritability is augmented' in both these limbs, and that this augmentation is most considerable in the limb whose nerves had been divided—in the limb, that is to say, which had been cut off from the spinal cord.*" (p.p. 60—63.)

Even the action of the will upon muscle is not allowed to be that of a stimulus :—

"Is it not certain," says Dr. Radcliffe, "that the will acts in this case by stimulating the muscle to contract? It is difficult, no doubt, to think differently. It is more than difficult to wean the mind from so old an idea. But, on the other hand, it must not be forgotten that the will may have *acted* in bringing about muscular contraction, not by imparting anything to the muscles, but by withholding something from the muscles; and this being the case, we may well refuse to allow a mere opinion respecting the action of the will, however sanctioned by time that opinion may be, to rank as an objection to a view of the action of 'nervous influence' in muscular motion, which view appears to arise necessarily out of the general history of 'nervous influence' as concerned in muscular motion." (pp. 64, 65.)

The action of blood upon muscle is considered after the action of nervous influence; and here the conclusion is, that contraction is not the result of stimulation. Many experiments are cited in favour of this view, and one of them will serve as an example. This was performed by Dr. Brown-Séquard upon the arm of a criminal who was guillotined on the 12th of July, 1851, at eight A.M. :—

"This arm, which was severed from the body, was in a state of perfect *rigor mortis* at 11 P.M.—fourteen hours after decapitation—and at this time the experiment was commenced by injecting a pound of defibrinated dog's blood into the brachial artery. As the blood began to penetrate into the vessels, some reddish spots appeared in different parts of the skin of the forearm, of the arm, and more particularly of the wrist. Then these spots became larger and larger, and the skin acquired the appearance it has in rubeola. Soon afterwards, the whole surface had a reddish-violet hue. A little later still, and the skin had acquired its natural living colour, elasticity, and softness, and the veins stood out distinct and full as during life. Then the muscles relaxed, first the fingers and lastly the muscles of the shoulders, and on examination they were found to have recovered their lost irritability. At 11.45 P.M. the muscles were more irritable than they had been at 5 P.M., at which time the corpse was first examined; and this degree of irritability was kept up, without abatement, until 4 A.M., when fatigue compelled Dr. Brown-Séquard for the time to abandon the experiment. When the experiment commenced, the temperature of the blood was 73° Fahr., and that of the room 66° Fahr." (pp. 67—68.)

After relating these experiments, Dr. Radcliffe proceeds to combat Dr. Brown-Séquard's theory, that muscular contraction is caused by the stimulus of carbonic acid in the blood, that is, by venous blood :—

"It may, indeed, be questioned," he says, "whether the convulsions of asphyxia are not rather due to the want of the stimulus of red blood than to any stimulus derived from the black blood with which the system has become charged; for it is certainly true that the muscles are similarly convulsed when an animal is bled to death. In other words, it is certainly true that the muscles are similarly convulsed, as well when the animal is left without blood as when it is left full of venous blood.

"Nor can it be allowed that the (apparently) more powerful contraction of the left ventricle during the first moments of asphyxia are due to increased stimulation on the part of the venous blood; for the fuller and firmer pulse, and the rise of the mercury in the hæmadynometer may be owing, not to increased contraction in the ventricle, but simply (as is indeed allowed on all hands) to the fact that there is some impediment to the free flow of blood through the capillaries of the systemic circulation—an impediment, that is to say, by which the systole of the ventricle is made to expend itself with greater force upon the coats of the intermediate arteries.

"Again, it may be questioned whether the muscular contractions which are produced in the two other experiments, when black blood is injected into the vessels, may not also be due to the want of some stimulus belonging to the red blood rather than to the action of any stimulus derived from the venous blood. At any rate it is well known that the uterus has often contracted and expelled its contents when a pregnant animal has been bleeding to death. And there are several facts on record in which the human uterus, even, has expelled its burden after the mother has yielded to the utter syncope of actual death.

"As it seems, however, the grand difficulty in the way of accepting this idea—that muscle is stimulated to contract by venous blood—is a chemical difficulty. For what is the main difference between arterial blood and venous blood? It is that the oxygen of the former has in the latter become displaced by carbonic acid. Now, carbonic acid has an action upon all parts of the nervous system which minister to intelligence or sensibility—upon all parts of the frame indeed, with the supposed exception of those which minister to motion—which action is so obviously opposed to that of stimulation, that it is extremely difficult to suppose that carbonic acid can be a stimulus in any case. Indeed, it is so difficult, as to make it well nigh impossible to entertain such a supposition for a single moment."—(pp. 74—76.)

The next point in the argument is to show that muscular contraction is not produced by the stimulation of any mechanical agent. This section of the argument is not altogether satisfactory. The idea is, that the needle, or any other mechanical agent which has provoked the contraction, may have served to *discharge* the electricity with which the muscle is in a manner charged, and that contraction may be the result of this discharge; and, certainly, some difficulties in the way of this hypothesis are disposed of with considerable cleverness. After this, it is shown that the history of muscular contraction, as seen in the vesiculæ seminales, or bladder, or bowel, or uterus, is at variance with the idea that this contraction is called into existence by anything like mechanical stimulation. The case of the uterus must serve as an instance of the way in which our author treats this part of his subject:—

"And, certainly, the doctrine of stimulation is not wanted to explain the parturient contractions of the uterus. At the time of labour this organ returns from the state of progressive expansion in which it had been kept during the period of pregnancy; and as *one* cause of the previous state of expansion would seem to be found in the increasing vital activity of the fœtus, so now *one* cause of the return from this state would seem to be found in the failure of this activity—a failure brought about, first in the mother, and afterwards in the fœtus, in consequence of the growth of the fœtus having then passed the limit beyond which it cannot pass without trenching upon the supplies necessary for the proper nourishment of the mother. It would seem, also, that this return of the uterus from the expanded state, or, in other words, this contraction of the uterine walls, must compress the vessels going to the placenta,—that the vital activity of the fœtus must suffer a corresponding depression from this interference with the sufficiency of the placental respiration—and that this depression must again lead to contraction in the uterus—for if this organ contracted in the first instance in consequence of a depression of this kind, there is no reason why it should not do so again. And, further, it would seem that this second contraction must lead to a third, and the third to a fourth; and that thus, the uterus acting upon the fœtus, and the fœtus reacting upon the uterus, contraction must follow contraction, until the completion of birth. Nor does it follow from this hypothesis that the uterine contraction should be unintermitting, for it is quite possible (this among other reasons) that the blood which is displaced from the uterus during contraction may temporarily 'stimulate' the system of the mother to a degree which is inconsistent with an unintermitting continuance of contraction in any of the muscles belonging to the involuntary system. At any rate, it is quite impossible, upon any rational view of parturition, to refer the contraction of the uterus to any 'stimulation' on the part of the fœtus, without ignoring the whole of the previous history of pregnancy."—(pp. 86—88.)

That muscular contraction is not produced by the stimulation of *light* is argued from the apparent expansion which takes place under this influence in the cushions of the sensitive plant, and in the iris. And, certainly, it is more easy to suppose, with Bichât, that the iris expands, and in this way closes the pupil, than to suppose that this closure is brought about by the action of sphincter fibres, which have a very doubtful existence.

Nor are we allowed to say that muscular contraction is produced by the stimulation of heat or cold; for the fact is, that the muscular current and nerve current—which currents, according to the premises, would seem to antagonize contraction—are annihilated or greatly weakened by that degree of heat or cold which is sufficient to throw the muscle into a state of contraction.

And, lastly, it is shown that muscular contraction is not produced by the stimulation of any chemical or analogous agency. Here a good deal of evidence is produced, and, among the rest, some recent experiments by Dr. Harley upon the action of strychnia and brucia, which seem to show most conclusively that these poisons act, first of all, by rendering the blood less apt to appropriate its stimulating element oxygen, and, in the second place, by diminishing the irritability of the muscles. Reviewing the whole evidence belonging to this part of the subject, there appears to be no sufficient reason why *rigor mortis* may not be taken as the type of muscular contraction in general:—

"For what," asks Dr. Radcliffe, "is the case with respect to this form of muscular contraction? The case is simply this: As long as there is any trace of that action of which the 'muscular current' is a sign, so long is there no *rigor mortis*. As long as there is any trace of that action of which the 'nerve current' is a sign, so long is there no *rigor mortis*. If this action dies out speedily, as in persons in whom the vitality of the frame has been exhausted by long life, or by chronic disease, such as consumption, the muscles become speedily rigid; if this action dies out slowly, as in persons who have been cut down suddenly in the full glow of life, the muscles are equally slow in becoming rigid. Once contracted, moreover, the muscles remain contracted until they break up in the ruin of final decay—an event which happens most speedily in the case where the muscle retains its physical integrity least perfectly. And this is precisely as it should be according to the premises; for according to the premises all that is necessary to the commencement of *rigor mortis* is the cessation of that action of which electricity is a sign, and all that is necessary to its continuance is the absence of this action and the physical integrity of the muscular fibre. In a word, it is possible to explain those unexplained and seemingly contradictory facts which constitute the distinctive features of that contraction into which the muscles pass after death; and hence *rigor mortis* may be accepted, not only as a type of muscular contraction in general, but as an *experimentum crucis* in favour of the proposition—that *muscular contraction is not produced by the stimulation of any contractile power belonging to muscle.*"—(pp. 99, 100.)

Having attempted to show that muscular attraction is not to be referred to the action of any stimulus upon any property of contractility inherent in muscle, the author's object is next to show "that muscular elongation is produced by the simple physical action of certain agents—electricity and others—and that muscular contraction is the simple physical consequence of the cessation of this action." In this section of the argument, a good deal is said to show that the peculiar changes of form in muscle, in contracting and in passing out of this state, and the law of contraction during life and after death, are susceptible of a purely physical explanation. As to the changes of form, it is shown, by the experiments of Mr. Joule, of Manchester, that a bar of iron, when it ceases to be magnetic, loses in length and gains in breadth, without undergoing any change of volume—precisely as a muscle does when contracting; and, consequently, the change of form in a muscle, under these circumstances, cannot be looked upon as a vital phenomena. After this, it is shown that certain facts, which at first sight do not appear to be altogether consistent with the physical mode of regarding the phenomena of muscular contraction, may be disposed of. It is shown, for example, how the diminished degree and diminished power of muscular contraction after death—how the loss of power as the muscle contracts upon itself, and how the waste of substance in proportion to the number of contractions—may be accounted for without supposing that the contractile power is a vital endowment.

Dr. Radcliffe's third and last object, in the physiological part of his work, is to show "that the special muscular movements which are concerned in carrying on the circulation—the rhythm of the heart, and those movements in the vessels which are independent of the heart—are susceptible of a physical explanation when they are interpreted upon the previous view of muscular action." Our space will not allow us to enter into this part of the argument, and a hint or two as to its character must suffice. Thus, when speaking of the action of the blood in producing the cardiac rhythm, Dr. Radcliffe says :—

"Upon any existing theory of muscular action it is more than difficult to understand why the *ventricles* remain distended with blood during the full half of the rhythmic period, if the *ventricular systole* is in anywise called into existence by the stimulation of the blood; but this fact is not altogether unintelligible if, on the contrary, it be supposed (as must be supposed upon the previous view of muscular action)

that the office of the blood will rather be to antagonize the systole and induce the diastole. Indeed upon this view the difficulty appears to be at an end, for according to it the ventricles are thrown into the state of diastole by the stimulation of the blood which has been injected into the coronary system of vessels, and they remain in this state until this blood has given up its arterial properties and so ceased to be stimulating. And certain it is that the different action of the ventricles in anæmia and plethora is calculated to strengthen this idea. Thus: in plethora the pulse (which is the direct test of the action of the ventricles) is full and slow; in anæmia it is small and quick. In the one case, that is to say, the ventricle fills to distension with rich blood and the systole is deferred—in the other case, the ventricle takes in a small quantity of poor unstimulating blood, and the systole follows with scarcely any delay. The facts, indeed, are the very opposites of what they would be found to be if the blood stimulated the ventricle to contract, for in that case the pulse must be small and quick in plethora, and full and slow in anæmia. But if, on the other hand, the blood provokes the ventricle to the diastole by causing elongation in the muscular fibres composing this chamber, then it is intelligible that the ventricle should dilate more fully, and the dilatation continue for a longer time, when the blood is rich and warm, as in plethora, than when it is poor and watery, as in anæmia."—(pp. 115, 116.)

"On realizing the phenomena of the heart's action more distinctly, it becomes even still more improbable that the systole of the ventricle is caused by any kind of stimulation, and of the blood more particularly. For what are the facts? At the systole the blood rushes through the coronary arteries into the coats of the heart, and the diastole of the ventricles is attendant upon this rush. And after the blood has remained in these coats until it may be supposed to have lost some of its arterial properties, then the systole returns. These are the simple facts; and thus if stimulation has to do with the phenomena at all, it is with the diastole and not with the systole.

"It appears, indeed, as if the *ventricular diastole* were due, partly to the force with which the blood is injected into the coronary arteries at the ventricular systole, and partly to the elongating, electro-motive effects of the arterial blood upon the cardiac fibres. It appears, also, as if the diastole of the ventricles were made to continue as long as the blood retained its arterial properties, and that the systole returned when the oxygen was exhausted and the arterial converted into venous blood. And thus, it appears as if the rhythm of the ventricles had a *part* of its explanation; for according to this view, so long as the proper blood continues to be supplied, and so long as the ventricle continues to be capable of responding to it, so long must the systole give rise to the diastole, and the diastole be followed by the systole.

"A little further examination will also serve to show that the *systole of the auricles* must be contemporaneous with the *diastole of the ventricle;* for this *systole of the auricles*, there is reason to believe, is, in great measure, the mere *falling in* of the auricular walls upon the sudden withdrawal of blood from the auricles by the diastole of the ventricles. There is reason for this opinion in the absence of valves at the mouths of the veins opening into the auricles, and the reason is obvious. For if the auricles had to contract primarily like the ventricles, is it not fair to assume that there would have been valves to prevent the reflux of the blood from the auricles into the great veins? And if so, then there is no difficulty in accounting for the rhythm of the auricles; for the *auricular diastole*, which is virtually coincident with the ventricular diastole, will be partly due to the same cause as the ventricular diastole—namely, the rush of blood into the coronary system of arteries—and partly to the onward current of blood which is continually setting in from the veins; and the *auricular systole* will be *mainly* due to the collapse of the auricular walls upon the sudden passage of blood into the ventricles at the ventricular diastole."—(pp. 117—119.)

. . . . "And if, under ordinary circumstances, the blood acts in this manner, then there appears to be no great difficulty in understanding how it is that the heart, or a portion of the heart, may go on pulsating after removal from the body. For why should oxygen dissolved in the blood act differently from oxygen diffused in the air; why, for instance, should not the air, which bathes the surface and permeates every interstice, provoke a diastolic state in the separate heart or a fragment of the same, by rousing that polar condition which in the nerves and muscles is designated under the name of the nerve and muscular currents? Why may not the systolic state supervene upon this diastolic state when the polar condition fails, in consequence, as it were, of the arterial air having become converted into venous air? And why, again, should not the diastole return after every systole, so long, that is, as the muscle is capable of responding to the action of the oxygen, for it may well be supposed that the commotion of the systole will displace the venous air and bring the muscular and nervous tissues into relation with fresh quantities of arterial air? Assuredly there is no evident reason to the contrary, and there is one reason why this view should be received, and this is to be found in the fact that the rhythm is rendered more rapid, and even revived for some time after its actual cessation, by placing the heart in oxygen instead of atmospheric air, and that it is brought to a stop by placing the heart in a vacuum or by immersing it in hydrogen or nitrogen or carbonic acid." —(pp. 123, 124.)

Passing from the domain of physiology to that of pathology, Dr. Radcliffe proceeds to consider the conditions in which muscular contraction is in excess; namely, the different forms of tremor, the different forms of convulsion, and the different forms of spasm. In doing this, the interparoxysmal condition, the paroxysm, the appearances after death, the pathology, and the results of treatment are all passed in review, and the conclusion arising out of each part of the inquiry is found to be in harmony with the previous view respecting the physiology of muscular action. In no part is it found that tremor, or convulsion, or spasm, is brought about by excess of stimulation, actual or relative. We are altogether unable to follow Dr. Radcliffe step by step in a subject so extensive, and all we can attempt to do is to give a hint or two as to the character of the argument, and refer to the book itself for further information. These hints, we think, may be best taken from the parts where the pathology of tremor, or convulsion, or spasm, is deduced from a consideration of the phenomena connected with the vascular system. We think that these hints may be best taken from these parts; for, seeing that the functional activity of the system is in direct relation to the supply of arterial blood, it is to be expected that the key to the whole problem will be found in the condition of the circulation. Thus, the supply of nervous influence to the muscles is proportionate to the activity of the circulation of arterial blood in the nervous centres and nerves, and this supply cannot properly be kept up when the circulation flags. What then is the condition of the circulation in those cases where muscular contraction is in excess? The answer furnished by Dr. Radcliffe is, that this condition is always what it ought not to be if the muscles were over-stimulated at the time. In *simple epilepsy*, for instance—

"The frigid and clammy hand, the foot that will scarcely keep warm before the fire, the pale and sallow or dark and venous complexion, the habitual feeling of chilliness, are facts which appear to show that the circulation is wanting in vigour; and this inference is fully borne out by the pulse, which in simple epilepsy is rarely otherwise than weak and slow. Plethora, in the form so often exhibited in the butcher, is never met with, and feverish reaction is a rare occurrence, even as an accident. There are, indeed, cases of *epileptiform* disease, in which the circulation may exhibit a greater degree of activity; but these cases, as will be seen in the proper place, present no real objection to the conclusion that the habitual state of the circulation in epilepsy is one of depression.

"Nor is there any evidence of a contrary character in the fit itself. Upon the eve of the fit, if any change can be perceived, it is in all probability one in which the skin has become paler and more dusky than usual, and the pulse more feeble. At the instant of the fall, in many, if not in all instances, a corpse-like pallor overspreads the countenance —a phenomenon which can only be explained on the supposition that syncope is impending; a moment or two afterwards, a dull-red flush, rapidly deepening into livid blueness, or even blackness, takes the place of the paleness which had first overspread the countenance, the face and neck become frightfully bloated, and everything shows plainly that all respiration is at an end. This state of suffocation continues during the whole course of the convulsion, and so perfect is it, that little or no arterial blood can find its way into the arteries during the convulsion.

"There is, however, one fact which may be thought to show that there is an increased injection of arterial blood into the vessels during the convulsion. Such injection is evidently very imperfect at the onset of the fit—in many instances at least; for upon no other supposition can we explain the corpse-like paleness of the countenance, and the feeble and silent pulse at the wrist. This is evident. But if the finger be kept upon the wrist, it may be found that the pulse may rise during the convulsion, until it has acquired a force and fulness which it never had in the intervals between the fits; and if the hand be placed over the heart at this time, it may be found that this organ is beating tumultuously and with great violence. It may also be found that these signs of vascular excitement will continue for some time after the convulsion is over. These facts are evident and unmistakeable, but they do not show, as they might seem to do at first sight, that more *arterial* blood is injected into the arteries at this time. On the contrary, they necessitate a totally different conclusion when they are subjected to a strict scrutiny.

"Now it cannot be doubted, that the effect of cutting off the access of air to the blood is to prevent the free passage of the blood through the pulmonary capillaries, and to overload the right side of the heart and the venous system generally, at the expense of the left side of the heart and the arteries springing from it. In this way the right side of the heart may become so much distended that the auriculo-ventricular valves are separated, and the beatings of the ventricle are made to tell as much in driving the blood back into the veins, as in sending it onwards into the lungs. But it is not right to suppose that the arteries are empty. If, for example, the carotid of a rabbit be exposed, and a ligature placed around the windpipe, it is found that the blood continues to flow through the vessel, that the originally scarlet colour becomes darker and darker, until at last it is as black as that of the blood in the neighbouring jugulars. Two minutes to two minutes and a half are occupied in this transformation of the scarlet into black blood. It is found, also, that this black blood will escape from the cut vessel in as full a stream and with as much force at the expiration of two minutes or two minutes and a half from the commencement of the process of suffocation, as it did before the aëration of the blood was at all interfered with. Nay, it is even found that at this time the black blood will escape with greater force and in a fuller stream than it did when it was red; for on fitting a hæmadynometer into the vessel and testing the force of the pulse-wave before and after the tightening of the ligature upon the windpipe, the mercury in the instrument is seen to rise to a higher point than that to which it rose previously. Indeed, at this time it is evident, without the aid of any instrument, that the artery is more distended and more tense than it was before. This phenomenon is explained by the late Professor John Reid, who has investigated the condition of the circulation in suffocation more carefully and successfully than any other observer, as the result of an impediment to the free passage of the black blood through the systemic capillaries similar to that which prevents the free passage of the same blood through the pulmonic capillaries; and it is more easy to entertain this view, and to suppose that, in consequence of this impediment in the systemic capillaries, a greater proportion of the force of the left ventricle is expended in distending the arteries, than to suppose that the ventricle is 'stimulated' to increased action under these circumstances. And, lastly, as explaining the peculiarity of the pulse in suffocation, it is to be remembered that the blood is sent along the arteries with greater force and increased velocity during violent attempts at *expiration*, and that the pulse becomes soft, feeble, and less frequent during violent attempts at *inspiration;* and hence it may be supposed that the increased fulness and force of the pulse during the suffocation of epilepsy may be owing partly to the fact that during the whole of this time the air is prevented from entering the chest by the firm spasm of all the muscles concerned—a state which may be compared to that which obtains in forced and prolonged expiration.

"Hence, the increase in the strength and fulness of the pulse which may take place during the convulsion of epilepsy is no proof, as it might appear at first sight, that the brain as well as the rest of the system is at this time supplied with an increased quantity of arterial blood; for the black and bloated face and neck, and the absolute suspension of the respiratory movements, show most clearly that the pulse is then filled, not with red blood, but with black.

"After the convulsion there is little to notice in the circulation. When the convulsions cease, the respiration is speedily restored, and the re-admission of arterial blood into the system may be attended with some transitory and inconsiderable febrile reaction; but this reaction has nothing whatever to do with the convulsion, for when it appears the convulsion has departed, and if the convulsion returns it is not until the reaction has first taken its departure.

"Arguing, therefore, from the corpse-like paleness and comparative pulselessness of the onset of the paroxysm, and from the signs of positive and unmistakeable suffocation by which this stage of paleness and pulselessness is succeeded, the only conclusion would seem to be that the convulsion of epilepsy is connected with the want of a due supply of arterial blood. Indeed, the whole history of the paroxysm, as deduced from the condition of the vascular system, would seem to show that there is something utterly uncongenial between epilepsy and anything like arterial excitement."—(pp. 156—161.)

Nor is the case different in *epileptiform convulsion* connected with certain diseases of the brain — chronic softening, chronic meningitis, tumour, induration, hypertrophy, atrophy, congestion, apoplexy, inflammation—with fevers, with certain suppressed secretions, with irritation in the gums and elsewhere, and with the moribund state. In each case, on examination, the condition of the circulation is always the reverse of what it ought to be, if the muscles were over-stimulated at the time. In *inflammation of the brain*, for instance:—

"In *inflammation of the brain* the condition of the circulation is not uniformly the same at all times, but this condition, as will be seen by reflecting on what has been already said, varies little with respect to the convulsion.

"*Simple meningitis* begins with paleness of the skin, a feeble depressed pulse, cutis anserina, vomiting, rigors, perhaps convulsion. Then follow rapidly the symptoms of high febrile reaction and cerebral inflammation—the pulse becoming hard and frequent, the breathings irregular and oppressed, the skin, particularly the skin of the head, hot and burning. These symptoms of high febrile reaction and inflammation continue for two or three days, and then give place to an opposite state of things, in which the pulse loses its force and becomes weak, small, irregular, and the breathings are interrupted with frequent sighs and pauses. Or if at this time the pulse retains any degree of resistance, it is evident from the dusky colour of the skin and the suspirious and laboured respiration, that the whole of this resistance is not due to the injection of arterial blood into the artery. Now it is in this stage of collapse which follows the period of inflammatory and febrile excitement, or else in the stage of collapse which precedes the febrile and inflammatory excitement, and never during the actual period of excitement, that the convulsion happens. And this rule is constant. Indeed, the history of simple meningitis shows most conclusively that vascular excitement is as incompatible with convulsion as it is with rigor or subsultus.

"In *tubercular meningitis* the pulse is weak and variable from the very first, now quick, now comparatively slow, rising in frequency when the head is raised from the pillow, and falling upon lying down again; and from the very first, the respiration is irregular, unequal, and interrupted with frequent sighs and pauses. For some time there may be some little disturbance of a hectic character, particularly in the evening; but this soon comes to an end, and the prostrate pulse forgets to put on even this faint semblance of fever. In some cases, there may indeed be a short stage of fever, and something like cerebral inflammation, especially in young children; but as a rule, the symptoms are altogether of a passive, non-febrile, non-inflammatory character. In any case, however, the convulsion is connected with an extremely depressed state of the circulation, and never with a state of febrile and inflammatory excitement, if there be such a state.

"In *rheumatic meningitis*, also, there is little or no febrile excitement from the beginning, and the pulse has become feverless and utterly weak before the convulsion happens.

"In *general cerebritis*, the pulse, at first slow, soon becomes variable and readily affected by changes of posture: the respiration, also, is very variable and suspirious. From the first, indeed, there is scarcely any fever, and little heat of head, except the phenomena of cerebritis are mixed up with those of simple meningitis; but if such symptoms are present, they soon pass off, and give place to symptoms of slow sinking—a state in which hour by hour the breathing is more interrupted with sighs and pauses, and the pulse more powerless, unless it may derive some fictitious power from the difficult circulation of imperfectly arterialized blood, in which case the dusky countenance and the purple lips will show very clearly that the vessel is not altogether filled with arterial blood.

"In *partial cerebritis* there is even less febrile disturbance than there is in general cerebritis, and at no stage of the malady is there anything like increased vascular action."—(pp. 309—311.)

Such is a free sketch of the argument by which Dr. Radcliffe attempts to show that convulsion in all its forms—tremor, convulsion proper, and spasm—must be looked upon in a very different light to that in which we have been in the habit of regarding it. The subject is one of great importance—of special importance in psychological medicine; for is not convulsion a most common symptom of disorder of the brain? The subject is one also which claims immediate, as well as serious, attention; for if Dr. Radcliffe is right, the natural conclusion is, that the more appropriate means of treatment will be, not those which are calculated to calm excessive stimulation, but those which will sustain and rouse the powers of a flagging system.

---

